# Dog-Assisted Therapy in Mental Health Care: A Qualitative Study on the Experiences of Patients with Intellectual Disabilities

**DOI:** 10.3390/ejihpe14030036

**Published:** 2024-02-29

**Authors:** Anke van Schooten, Nienke Peters-Scheffer, Marie-José Enders-Slegers, Inge Verhagen, Robert Didden

**Affiliations:** 1 Center for Mild Intellectual Disability and Psychiatry, GGZ Oost-Brabant, Kluisstraat 2, 5427 EM Boekel, The Netherlands; ipc.verhagen@ggzoostbrabant.nl (I.V.); robert.didden@ru.nl (R.D.); 2 Behavioural Science Institute, Radboud University, P.O. Box 9104, 6500 HE Nijmegen, The Netherlands; nienke.peters-scheffer@ru.nl; 3 Driestroom, P.O. Box 139, 6660 AC Elst, The Netherlands; 4 Faculty of Clinical Psychology, Open University, P.O. Box 2960, 6419 AT Heerlen, The Netherlands; marie-jose.enders@ou.nl; 5 Institute for Antrozoölogy (IVA), Uilecotenweg 3, 5324 JT Nijmegen, The Netherlands; 6 Trajectum, Dokter Stolteweg 17, 8025 AV Zwolle, The Netherlands

**Keywords:** dog-assisted therapy, animal-assisted therapy, animal-assisted interventions, animal-assisted services, adults, mild intellectual disability, borderline intellectual functioning, mental health care, qualitative study

## Abstract

(1) Background: Dog-assisted therapy (DAT) is an experiential intervention to promote psychological, physical, and social functioning in children and adults. Only few studies have been conducted on DAT in adults with a mild intellectual disability or borderline intellectual functioning (MID-BIF). The purpose of this study was to explore the experiences of patients with MID-BIF undergoing DAT in a mental health care facility. (2) Method: Seven patients completed 13 to 15 sessions of DAT. Within two weeks of completing the program, they were interviewed using a semi-structured interview. The transcripts of the interviews were analysed using interpretational phenomenological analysis. In addition, the patients’ relatives and the DAT therapist were interviewed, and a focus group discussion took place with each patient’s treatment team. (3) Results: The patients’ experiences were predominantly positive. Physical contact with the dog calmed them down. The dog offered them emotional support and helped them to make contact inside and outside the therapy and the setting where they lived. The patients also liked the fact that DAT focused on the dog rather than their problems, that the therapy was experiential and using a positive approach, and that, during the therapy, they did not feel like a patient but a human being. DAT is a promising therapy for patients with MID-BIF in mental health care facilities, but more research into its effectiveness and cost-effectiveness and ways to implement DAT in clinical practice is needed to make more definitive statements.

## 1. Introduction 

Patients with a mild intellectual disability or borderline intellectual functioning (MID-BIF; IQ 50–85) are overrepresented in mental health care, with a prevalence rate between 20 and 43% in acute care [1]. According to the *Diagnostic and Statistical Manual of Mental Disorders* (DSM-5-TR), intellectual disability is defined by deficits in intellectual functioning and adaptive behaviour that must have originated during the developmental period. Patients with MID have an IQ score of 50–70, while patients with BIF have an IQ score of 70–85. Many patients with MID and those with BIF (if the latter also exhibit deficits in adaptive skills) require professional support. Combining MID and BIF levels is relatively common because these patients face similar challenges and have similar support needs, thus requiring similar treatment interventions.

Verbal and cognitive-oriented therapies may not fully consider the limitations of patients in terms of working memory, language, mentalization, and emotion regulation skills nor their difficulty in generalizing what they learn in therapy to everyday life. Experiential interventions, which focus on experiencing feelings, thoughts, bodily sensations, and behaviours rather than discussing situations outside therapy sessions, fit well with the characteristics and learning style of patients with MID-BIF [2]. Animal-assisted services (AAS), the umbrella term which was recently introduced to replace the term animal-assisted interventions (AAI) [3], are considered experiential interventions, where an animal is used for therapeutic purposes in the treatment or support of humans. Working with a dog is often chosen in AAS because dogs have the ability to adapt to human behaviour and understand and respond to (body) language. In addition, dogs are easily trained and available [4,5,6]. AAS in the form of therapy with a dog is called dog-assisted therapy (DAT).

There are several hypotheses and theories about how the interaction between dogs and people can have a positive impact on human functioning. The first hypothesis concerns the buffer and distraction that animals can provide for people in stressful situations, as calm animal behaviour can evoke feelings of support and safety in human beings. Feelings of relaxation are described by both pet owners and participants in a study where students were given the opportunity to be with pets while waiting for their exams to start [7,8]. As for the second hypothesis, interaction with a dog stimulates and facilitates contact between people [9,10]. The third hypothesis relates to the support people can experience from the mutual connection and attunement between humans and animals [11,12]. An increasing number of studies on DAT in patients with an average intelligence with mental health problems have shown that DAT is a promising intervention for improving social-communicative skills [13,14] and physical well-being [15] and reducing symptoms of depression [16], anxiety [17], and those related to trauma [18,19]. In acute mental health care for patients with an average intelligence, beneficial effects have been found on anxiety, depression, pain, and heart rate [20] as well as on the negative symptomatology of schizophrenia, cortisol levels, and treatment motivation [21].

To date, only few studies on DAT in adults with intellectual disabilities (including those with MID-BIF) and mental health problems have been carried out. A study on DAT in adults with severe intellectual disabilities has indicated a positive impact on social and communication skills, motor coordination, and activity levels [22]. Furthermore, in a study on adults with a mild intellectual disability, the participants rated lower self-report scores on anxiety after 30 min sessions during which they could play with, pet, or brush a dog [23]. 

In children and adolescents, some more studies on DAT have been carried out. Studies in children and adolescents with MID-BIF and mental health problems show that DAT leads to improvements in behavioural, cognitive, social, and emotional functioning [24,25,26,27]. The present study builds upon prior research by exploring the viewpoints on and experiences of DAT in adult patients diagnosed with MID-BIF. Specifically, it examines the perceived effects of DAT on their mental health and daily functioning. This study aims to identify the elements of DAT that contributed to its suitability and feasibility for these patients. Furthermore, it investigates whether the elements mentioned by the patients align with the pathways of impact mentioned in the literature on DAT. To this end, seven patients with MID-BIF, who stayed in a mental health care setting because of mental health problems, participated. In addition to interviews with these patients, their relatives, DAT therapist, and treatment team were interviewed about the feasibility and outcomes of DAT in these patients.

## 2. Materials and Methods

### 2.1. Patients and Setting

This study involved seven adults with both MID-BIF and mental health problems. The participants were between 27 and 48 years old, with a mean age of 35 and a standard deviation of 7. The sample consisted of four males and three females, who all had a Dutch nationality. One patient had a North African cultural background, and another had emigrated from the Middle East to the Netherlands at a young age. Three patients had primary education as their highest level of education, while four patients finished pre-vocational secondary education. The patients had comorbid mental health problems, including psychotic disorders, post-traumatic stress syndrome (PTSS), anxiety, mood disorders, personality disorders, and/or relapse in substance use.

In general, all the patients had had positive experiences with dogs in the past; however, one patient had developed some fear of unknown dogs after a negative experience with a dog. The patients aimed to improve their communication skills, attention and concentration, self-confidence, and stress regulation during DAT and wanted to reduce their (social) anxiety.

They voluntarily received 24 h care for an extended period of time in an open treatment unit located on the grounds of a mental health care facility in the south of the Netherlands. The ward housed sixteen men and eight women aged 24 to 70 years, all of whom had been diagnosed with MID or BIF and comorbid mental health problems. In addition, somatic problems were common. Treatment in this ward, which was aimed at recovery and rehabilitation, was provided by a treatment team consisting of nurses, psychologists, and a psychiatrist. The daily program had a more or less fixed schedule and consisted of group activities indicated for each patient and group as well as individual treatments.

All the patients, their relatives, and the treatment team gave written consent to participate in this study. This study was approved by the Science Committee of GGZ Oost-Brabant and the management of the facility. After ethical review, approval was issued by the Social Sciences Ethics Committee of Radboud University (ECSW-2022-081).

### 2.2. Dog-Assisted Therapy

The DAT program in this study was based on the DAT program for children with intellectual disabilities and adults with autism spectrum disorders [10,11,28]. To ensure the quality of the intervention and the safety of patients, dogs, and therapist, the DAT program was developed according to the guidelines of the International Association of Human-Animal Interaction Organizations [29] and the Guidelines for Quality Development and Quality Assurance in AAI [30]. The DAT therapist was certified and experienced in DAT and in working with patients with MID-BIF and mental health problems. The dogs, a male 4-year-old and a female 6-year-old black Labrador retriever, had been trained as therapy dogs, lived in host families, and came to the therapy site for up to two sessions of 45 min per day. Between sessions, they were cared for by a volunteer. The dogs were matched with patients by the DAT therapist based on their liveliness and responsiveness.

The DAT program consisted of 16 weekly 45 min individual therapy sessions. The sessions took place in both a therapy room and on the outside grounds of the facility. Therapy began after the patients were introduced to the DAT therapist and the dog on the ward and after an intake interview in which the treatment goals were determined.

During the first phase of the DAT program, sessions 1–5, the therapist focused on assisting the patient in establishing a connection with the dog, facilitating this connection through comments or questions regarding the dog’s behaviour. The patients were instructed to observe and interpret the dogs’ behaviour during free play and basic exercises, such as calling the dog to stand or lay down on its dog bed. In addition, the patients were taught to observe signs indicating the dog’s willingness to play or work with them and to verify that the dog had correctly understood and executed their commands. The DAT therapist provided subtitles for the dog’s behaviour and assisted the patient in observing their own behaviour in relation to the dog. The exercises were customized to the patient’s individual abilities and treatment objectives, with consideration given to the type, intensity, and level of independence required, and were assisted in a way that allowed the patient to complete them as independently as possible. For instance, the majority of patients expressed a desire to improve their ability to communicate their needs more clearly. By practicing basic commands with the dogs, the patients were able to reflect on their own behaviour in cases of no reaction or disobedience of the dog and were given the chance to practice an alternative behaviour with the dog until the exercise was successful. Additionally, the patient was provided ample opportunity to interact in a relaxing way with the dog, including petting and cuddling. This was also the case when the patient was feeling upset at the beginning of the therapy session or if either the patient or dog required relaxation between exercises. This helped bring the session to an end on a positive and calm note. 

During the second phase of the DAT program (sessions 6–10), the patients’ confidence in their ability to work with the dog and therapist was strengthened through repeated and expanded exercises in which new behaviour was learned. The patients who successfully led the dog through the therapy room took a next step by constructing a slalom course and guiding the dog around the pylons. If successful, the dog was directed along the course by the patients using only their voice and attitude. Depending on what the patient could cope with, a possible next step included relying solely on body language without verbal communication. In addition, the patients were taught to keep their dog under control while walking outside with the therapist, whether the dog was on or off the leash. The patients were instructed to reward the dog for each step taken or to stop and reassess the situation if the dog did not behave as desired. The therapist expanded the analysis from the dog’s behaviour to that of the patient. The therapist discussed with the patient which of their behaviours led the dog in the right direction or why the dog may have been confused. The patients learned that a lack of focus in the dog may not always have been their fault but could also have been caused by external factors such as noise or the dog being tired or mischievous. Moreover, the patients observed that the therapy dog did not always respond immediately to the therapist’s commands.

In the third phase of the program (sessions 11–16), the exercises were more explicitly linked to the specific treatment goals of a patient. For example, the patients who set goals for limit-setting were taught to train their dogs to stand still at a distance or walk to a predetermined spot and wait there, using body posture and spoken commands. These patients learned to communicate their commands in a clear and structured manner. In this last phase, the therapist assisted the patient in applying the acquired skills to their everyday life.

### 2.3. Procedure

This study comprises 16 interviews with the patients, their relatives, and the DAT therapist and one focus group conversation with the treatment team. Based on a recent systematic review on the required sample size in qualitative research, it is suggested that conducting 9–17 interviews or 4–8 focus interviews is sufficient to reach data saturation [31]. This statement is only relevant if the target audience is clearly defined and the research question is well-defined, as is the case in this study. Data saturation is defined as the point where no additional issues or insights emerge from data, indicating that themes are comprehensively and credibly conceptualized in the study context in question. Previous research has distinguished between code saturation and meaning saturation. Code saturation is said to be reached after nine interviews, indicating that no new themes emerge in interviews. Meaning saturation, on the other hand, is achieved after 16–24 interviews, when all relevant themes have been identified and thoroughly understood by the researchers [32]. 

By conducting interviews with not only the patients themselves but also their relatives, therapist, and treatment team, we were able to collect rich data that provided valuable context to the patients’ experiences.

The inclusion criteria for participation in this study were as follows: (1) a diagnosis of MID or BIF based on the results of intelligence tests and the Adaptive Performance Test (ADAPT) [33]; (2) treatment goals aimed at improving social skills and communication, concentration, attention, and motivation or decreasing stress, anxiety, and mood problems; (3) positive indication from the treatment team regarding the participation and completion of therapy and research; and (4) written consent from the patient and, if applicable, their legal representative. The exclusion criteria were the following: allergic reactions to dogs, violent behaviour toward animals, and extreme motor or behavioural impulse control problems. Seven patients were included who varied in age, gender, and type of mental health problems.

Due to circumstances (e.g., illness), the patients attended between 13 and 15 of the 16 scheduled sessions. Within two weeks after completion of the DAT program, the patients were interviewed in the therapy room or in the ward by the first author in the presence of the therapy dog with whom the patient had worked. A supplementary interview took place with two patients so that themes that emerged from the initial interview could be deepened with examples. One patient could not be interviewed because, after 13 DAT sessions, she left the treatment ward on her own initiative and stopped all contact with the facility. However, her legal representative was interviewed.

The semi-structured interview started with an open-ended question about experiences with DAT, after which the interviewer continued to ask about experiences regarding the content, atmosphere, and efficacy of the therapy. To adapt to the deficiencies in communication and reflective abilities, concrete examples of what happened between the patient and the therapy dog during the sessions were requested. Also discussed were the similarities and differences with other forms of treatment (e.g., cognitive behaviour therapy) and the changes in behaviour, thoughts, and emotions experienced by the patients and/or observed by those around the patient within and outside therapy in the context of the treatment goals they had set for themselves. Further questions were asked about the impact of the therapy dog on the patients and the collaboration with the DAT therapist. Finally, the patients were asked if they had any suggestions to improve the DAT program and if they would recommend DAT to other patients. As a thank you for their participation, the patients received a framed picture of the dog. The interviews lasted from 30 to 60 min, with the exception of one 10 min interview with a patient.

The patients’ experiences were contextualized by discussing them with their relative, the DAT therapist, and the treatment team after therapy. From an open-ended question about what the relatives had noticed about their child, brother, or sister, experiences and reflections on the therapy were asked in the context of the relatives’ perceptions of the patients’ strengths and vulnerabilities and their ideas about what it was like for the patients to work with the dogs. During the focus discussion with the treatment team and the interview with the DAT therapist, the patients’ experiences were tested for recognizability and for positively labelled change or negative effects of the therapy.

### 2.4. Data Analysis

The interviews and discussions were audiotaped and transcribed. The patients’ names were anonymized using a pseudonym. The data were analysed using interpretational phenomenological analysis (IPA) [34], as IPA provided the researchers with the opportunity to interpret the patients’ thoughts, feelings, and experiences and connect them to the scientific literature and the experiences and reflections of the relatives, therapist, treatment team, and research team. The IPA method appears to be appropriate for people with MID-BIF [35,36,37]. IPA uses a dual mode of hermeneutic reasoning: a patient uses the interview to reflect on and make meaning of his or her experiences through the questions asked by the interviewer. Researchers then interpret the patient’s interpretations during the analysis of the interviews, both in the context of their own experiences and beliefs and the collective making of meaning by all patients, their loved ones, and the treatment team [38].

The interviews were analysed in rounds of two interviews, following three steps in each round. In the first step of data analysis, the first, second, and fifth author (AvS, NPS, and RD) read all the transcripts of the interviews, with relevant excerpts being marked independently by the three authors. The excerpts were then coded, and salient features were noted. These were discussed, and codes were grouped based on the saturation of information and the delineation of themes.

An interview rich in reported experiences and the interviews with the treatment team and with the DAT therapist were read by the third author (MJES). From her expertise in AAS, she contextualized these experiences. During the DAT program, logs were kept by the DAT therapist and the main researcher. The logs were read by the fourth author (IV) and placed in contextual relevance in the field of treatment of patients with MID-BIF and mental health problems.

## 3. Results

During the interviews, the patients talked about both the role of the dog in the therapy and the therapy itself. The analyses of the interviews yielded seven themes: physical contact with the dog, emotional support inside and outside therapy, making contact with people via the dog, “the dog is the focus instead of my problems”, experiential character of DAT, DAT as a positive therapy, and DAT helps patients to feel normal.

### 3.1. Physical Contact with the Dog

“When I had re-experiences, I would come in sad and tired. Then I would sit with the dog, and I would calm down. One time the dog licked the tears off my cheeks, which made me laugh. Then we started to work with the dog as we always do. I felt much better when I left after the session.”

Chantal

“I personally found that I became calmer in my body when I was working with the dog.”

Peter

“I don’t feel alone when my feet get warm because the dog lay down on them.”

Ingrid

The patients had experienced many adverse events in their past, which made them often tense in their daily lives. During the interviews, they mentioned that they felt like having “a busy head” and “a restless body”. In the focus group, it emerged that the patients had been in a residential setting for a long time and that they had hardly any physical contact with other people and animals. During DAT, the patients were able to pet the dog and play and cuddle with the dog. All the patients mentioned feeling calm and comforted by the physical contact with the dog. At times when they were tense and/or the interview seemed to be stalling, the patients sought physical contact with the dog by sitting on the ground with the dog, calling the dog to them and petting it, or chatting with the dog in between.

### 3.2. Emotional Support Inside and Outside the Therapy

“The dog recognizes me and stands at the door wagging her tail when I approach…. she always wants to work with me, even when I’m sad or tired. When I was anxious, it was like she—yes, that sounds stupid—she knew. Then she would look at me with those little eyes like: I’m staying with you. At first I thought the dog didn’t like me, but she knew after just one session that I was coming to work with her. […] I have a picture of the dog on my phone. I look at the picture when I am sad. […] I dare now to stay home alone when I am with my mom, because mom’s dogs are with me.”

Chantal

“The dog understands me. I can tell by the look in his eyes when he looks at me. I like that feeling.”

Leo

“Many people with whom I had a connection are gone. The dog is there for me and because of that I know that a good heart exists.”

Mohammed

The patients had a small social network and had little contact with others. In their interviews, all the patients told the researchers about the bond they had with the dog during DAT. They mentioned that the dog was a companion for them, recognized them, understood them, and was happy to work with them, even when they were sad or tired. The dog did not judge them, which also stimulated the patients to go to DAT even when they were not feeling well, because they enjoyed seeing the dog again. For example, Marcel said, “I come every session, I don’t leave him [the dog].” Outside DAT, some of the patients also sought more frequent contact with dogs around them. For example, Chantal now dares to stay home with her mother’s dogs when her mother runs an errand, whereas before she did not dare to stay alone in the house.

### 3.3. Making Contact with People via the Dog

“The therapist is kind and cheerful to the dog so I think she is a sweet woman […] I used to walk away when I got scared. With the dog, I learned to face the situation. A dog is different from a human, a dog is real and less complicated. It won’t cheat on you. Now, we [the therapist and I] chat and laugh about the behaviour of the dog and about ourselves.”

Gemma

“I noticed that people like seeing the dog. They make contact. I dared to look at them, which I never do otherwise because I am afraid they will be angry with me. I saw now that they were friendly to the dog. Then I also acted friendly. I also say ‘hi’ to people now when I am alone and I dare to look around me.”

Mohammed

“About other therapies we never hear anything, but about working with the dog she doesn’t get tired of talking about it.”

Mother of Chantal

The focus group revealed that the patients generally found it difficult to make contact with others and trust others. The patients said that the dog helped them make contact with the DAT therapist because conversations would arise about (the behaviour of) the dog but also because they saw that the therapist was nice to the dog and they deduced from this that the therapist would also be nice to them and could be trusted. For example, during a therapy session, one patient mentioned that she had been in an abusive relationship for quite some time. She had not talked to anyone about this until she told the DAT therapist about her secret. With the therapist’s support, she was then able to share it with other members of her treatment team. In the focus group, the treatment team called it remarkable how quickly and how openly the patients told the therapist about their fears, compulsions, substance use relapses, pain from the past, and issues from everyday life.

Contact with other people was established as some of the patients walked with the dog and the DAT therapist outside the facility grounds. Outside the sessions, conversations with relatives, staff, or fellow patients were started, during which the patients shared their experiences with the dog and the DAT program. According to their relatives and the nurses of the treatment team, the patients liked and talked positively about the dog and the DAT program, and it was easier for them to start talking about DAT after the sessions.

### 3.4. The Dog Is the Focus Instead of My Problems

“Therapy starts with a happy dog, who wags when I stand at the front door, whom I go to pet and with whom I chat when I come in. That dog doesn’t ask “How are you?” I think that’s a rotten question, usually because I can’t say I’m doing well.”

Chantal

“At DAT you give commands and do tricks with the dog. If you’re not clear, then he won’t know what to do. And by learning to be clear to the dog, I also learned to be clear to people. […] If one time it doesn’t work out, it’s not necessarily because of me or the dog. Sometimes it’s because of the situation. I don’t pay as much attention to my thoughts of doubt anymore.”

Mohammed

“Normally, I have compulsions. During DAT I don’t, I’m just so busy with the dog.”

Peter

In the focus group, it emerged that, within the mental health care system, there is a lot of attention for complaints and symptoms of the patients and that, as a result, patients are focused on (negative aspects of) themselves. The patients mentioned that they liked the fact that, during DAT, they were focused on working with the dog and not on their problems. The playful and, to some extent, unpredictable behaviour of the dog during DAT meant that they had to keep their attention on what was happening in the therapy and that there was no room to focus on their complaints. For example, during DAT, Peter did not show any compulsory behaviours. 

In reflecting on the exercises, attention was paid to how the patients’ thoughts, feelings, and behaviour interfered with daily functioning and reflected on how alternative strategies that worked with the dog could possibly also work in daily life.

### 3.5. Experiential Character of DAT

“This therapy is not an hour of talking but an hour of working. We often repeat an exercise, then I see how much better and faster the dog listens to me.”

Mohammed

“The therapist explained and we made a plan for the exercise together. Then we set to work. We sometimes made a video of the exercise on my phone. Then we would look back and I would see what I could do differently. I did that and then I saw: oh this is how it works well, so to speak”.

Gemma

The patients experienced DAT as a therapy that involved working with the dog rather than talking about their problems and vulnerabilities. This type of “learning by doing” with a large focus and reflection on what happens here-and-now during the session seemed to fit the learning style of the patients. When they tried things out, they saw immediate results of their work: the dog listened well, a little, or not at all. Physically experiencing (alternative) behaviour in the here-and-now was supportive and encouraging for the patients, as well as discovering that learning happens in steps rather than that all steps should be learned at once.

### 3.6. DAT as a Positive Therapy

“I have to laugh all the time at the dog’s mischievous behaviour, not listening […] The therapist and I have come up with a name for the dog’s busy, stubborn behaviour: ‘wet nose behaviour.’ I have to laugh so much when he listens better to me than to the therapist, which happens sometimes!”

Peter

“DAT is just really interesting. At first I thought, ‘oh, that seems scary, I don’t want that,’ but it’s not so bad. The therapist said, try it with small steps then. You just have to try things. And if the dog doesn’t listen, you have to think: why is that?”

Mohammed

“I am proud of what I have achieved in therapy. I now know better what I like in communication and I also tell that to the treatment team. I notice that I can do things better when I feel calm, like in therapy. When I am tense, I often still find it difficult.”

Leo

The focus group revealed that, due to mental health problems, previous negative learning experiences, and limited success, patients have difficulty exploring new situations and challenges because they assume that they will not be able to handle them successfully. Normally, they spend considerable time brooding about things that do not go well. Nurses regularly came to a session with a patient if s/he had become overwhelmed with emotions earlier in the day but was nevertheless eager to go to DAT.

During the interviews, it emerged that the patients experienced the therapy as enjoyable. During the sessions there was a lot of laughter about the dog’s behaviour and what was not (yet) going well. They experienced that the failure of an exercise was not necessarily their fault: sometimes the dog was tired or the instruction was unclear. They also noticed that the dog did not always listen to the DAT therapist. Because of this, the patients noted that they had to “persevere” and “figure out what works for the dog”, such as communicating more clearly, calming down first, or asking the therapist for help when they were tired, restless, scared, or sad. The patients were proud that they managed to persevere and that they eventually performed the exercises successfully. 

According to the treatment team, such experiences contributed to the patients discovering that they could achieve more than they thought they could and that they enjoyed learning new things if it was performed in a way that suited them. The treatment team also noticed that, unlike in other therapies, the patients made great efforts to attend the therapy sessions, for example, by rescheduling their work and by making sure that they were sober in case of substance use.

### 3.7. DAT Helps Patients to Feel Normal

“When I walk with the dog and therapist, I feel so normal.”

Ingrid

“Here you just do an exercise. You get to put your own spin on it with the help of the therapist. […] I decide if the dog does well and then I’m the one who rewards the dog.”

Leo

“I normally can’t concentrate for long, but in therapy it comes naturally because I enjoy it so much.”

Mohammed

During the focus group it emerged that patients are often seen as patients, who receive 24 h care in a residential setting. In their life, they often experience that they do not understand situations well or cannot keep up with their peers, at school, and at work or day care. As Leo and Ingrid mentioned, during DAT, the patients did not feel different but rather “normal” because of the exercise they carried out with the dog, such as walking, playing, and cuddling. This feeling was enhanced by the fact that the therapy took place in a homely setting. They also noticed that, when they were with the dog, other people saw them not as a patient but as the dog walker and a normal human being. As a result, the patients dared to go out on the street. For example, Chantal mentioned how normal and protected she felt by the dog because the dog would bark if others got too close. The patients told the researchers how they were in charge during the exercises they performed with the dog, very much unlike their daily lives, in which they often depended on others. They took initiative and had influence and a say in what happened and how it happened during the DAT program.

## 4. Discussion

This study explored the experiences with DAT of seven patients with MID-BIF and mental health problems who lived in a mental health care facility. The experiences were generally positive. Through physical contact with the dog, the patients calmed down and experienced comfort. The dog offered them emotional support and helped them establish contact with other people inside and outside therapy. In addition, the patients liked the fact that, during DAT, the dog was the focus rather than their problems, that the therapy was experiential and positive, and that they did not feel like patients during therapy.

This study extends the small number of studies on DAT that have been conducted in adults with MID-BIF. By exploring the views of patients, their relatives, and the treatment team, we obtained rich information about their experiences with DAT [39]. These insights from the patients’ own perspective are considered essential because of the principle of “nothing about us without us”, which states that patients should be involved in policies and decisions which affect their health [40,41,42]. 

The experiences of the patients in our study are consistent with research on the decrease in stress markers during DAT in people with an average cognitive ability and mental health problems, both physiologically [43,44] and psychologically [45]. A study on DAT in adults with ASD described a significant reduction in cortisol levels, which supports the feeling of calm and relaxation [43]. Another study discussed the activation of the oxytocin system as an underlying explanation for the positive psychophysiological and psychological effects in both humans and animals during AAS [44]. A study on the impact of dog-assisted activities on inpatients with schizophrenia revealed positive outcomes in terms of self-esteem, self-determination, and a reduction in psychotic symptoms [45]. In addition, other themes have also been mentioned in studies on DAT, notably about the emotional support a dog provides during DAT [12] and the dog’s function as an icebreaker in the contact between people with and without intellectual disabilities [46,47]. These issues are also reflected in research on the (non)specific factors of AAS with a range of animals [48]. For example, in a sample of 16 adults with intellectual disabilities, it was found that a dog walking program, which was classified as a dog-assisted activity rather than DAT, facilitated encounters with other community members [49]. The number of encounters was significantly higher when they participants walked with a dog and its handler than when they went for walks with the handler alone. According to the patients, the presence of the dog helped in breaking social norms about speaking to strangers and discourage disrespect toward people with intellectual disabilities.

Positive contact and experiencing genuine interest are a prerequisite for successful therapy in general [50]. The patients in our study liked that the session started with a cheerful, nonverbal, and nonjudgmental greeting by the dog. The dog recognized them and responded to their mood and behaviour, while managing the dog during the sessions distracted the patients from their problems. The patients additionally drew positive conclusions about the DAT therapist because of the way in which she interacted with the dog. The exercises and the dog’s response to the patients’ behaviour met the needs of patients with MID-BIF to work “in the moment”, in an experience-oriented manner, with a limited use of language. The patients were able to remember the exercises well and, with the therapist’s help, practiced new skills in the therapy sessions, such as planning activities, carrying them out step by step, communicating clearly, and setting boundaries. The patients connected situations in their daily lives with the therapy and devised a plan with the therapist to practice new behaviours outside of therapy. The patients mentioned in the interviews that they needed help from the treatment team to practice new behaviours in daily life.

The focus group revealed that the patients usually had difficulties sustaining therapies. But the patients were quite positive about following the DAT program and mentioned this to the treatment team and their relatives. Moreover, the patients motivated other patients to participate in DAT. Other studies also found that shared enjoyment between patient, dog, and therapist and successfully managing the dog can evoke feelings of competence, enjoyment, and motivation [51,52] and reduce dropout of therapy [18,28].

Finally, the normalizing aspect of DAT was mentioned by the patients. Working with animals in the presence of a therapist or nurse has also been perceived as positive by people with mental health problems in other types of AAS, due to opportunities to carry out “ordinary things” and be distracted from illness [53].

The seven themes that emerged from the interviews and focus group reinforce each other. A study on theories and processes in AAT states that, when psychological and physiological stress responses are deactivated, intrinsic motivation is activated, and a situation of learning is created [15].

The implementation of the DAT program was challenging in terms of the time, money, and energy that working with live animals and their caretakers requires (also see guidelines of the International Association of Human-Animal Interaction Organizations [29]). The generalization of the positive outcomes of DAT to the daily lives of the patients was challenging as well. The patients needed active support to learn to apply skills such as setting boundaries or asking for help in new situations. Furthermore, the patients found it difficult to say goodbye to the dog, and, in this DAT program, the number of sessions was limited. 

During the research, we encountered limitations regarding the sample size. The DAT program was implemented for patients from one department due to the practical cooperation between the DAT team and the treatment team as well as the desired homogeneity of the target group. Consequently, the number of patients who could participate in the program and, therefore, the study was relatively small. 

While in line with the iterative principle of qualitative research, combining the project management and interviewer roles in one person carries the risk of attention and confirmation bias. It is important to separate these roles to ensure objectivity and avoid potential biases. To mitigate this risk, the first author maintained open communication with the co-researchers throughout the project and critically reflected on each step of the research during its preparation and implementation to ensure the collection of versatile data.

Although the patients were highly motivated to discuss their therapy and could provide concrete examples, they found it challenging to provide answers to the interviewer’s questions about how DAT affected their daily functioning and mental health, in line with their cognitive abilities. The patients found it difficult to determine how they would fare without DAT.

The treatment in this study was shaped from a DAT protocol, with room for the therapist to tailor to the patients’ needs and physical, social, and emotional abilities. As in other therapies, the quality of the therapist is essential [54,55]. In DAT, a therapist should have knowledge of dog behaviour and well-being and the delivery of therapy to the target population in question. Little has been studied about the treatment components of DAT, the role of the therapy dog, and how DAT can be embedded in the treatment program of mental health care facilities [24]. Although the patients, the relatives, and the treatment team reported positive effects, consistent with the pathways of impact from previous studies, we only identified observable effects in this study. 

Future research should be directed at evaluating the cost-effectiveness of DAT for patients with MID-BIF who reside in a mental health care facility. Furthermore, future research could investigate the long-term effects of dog-assisted therapy (DAT) and describe in-depth the therapy protocol and the therapist’s competencies and interventions. The experienced or measured differences between DAT and dog-assisted activities for different target groups could be studied. Additionally, future research could explore the practical aspects of DAT in mental health care, such as determining the optimal number of sessions and the role of the therapist in generalizing skills and strengthening positive beliefs in daily life.

DAT can contribute to a stimulating and dignified treatment process from its normalizing and positive character, its experiential approach, and the contact between patient, dog, and therapist.

## Data Availability

The data that support the findings of this study are available from GGZ Oost Brabant. Restrictions apply to the availability of these data, which were used under the license for this study. Data are available from the corresponding author, AvS, with the permission of GGZ Oost Brabant.
